# Nursing home administrators’ perspectives on a study feedback report: a cross sectional survey

**DOI:** 10.1186/1748-5908-7-88

**Published:** 2012-09-13

**Authors:** Anne-Marie Boström, Lisa A Cranley, Alison M Hutchinson, Greta G Cummings, Peter G Norton, Carole A Estabrooks

**Affiliations:** 1 Division of Nursing, Department of Neurobiology, Care Sciences and Society, Karolinska Institutet, Huddinge, Sweden; 2 Department of Geriatric Medicine, Danderyd Hospital, Danderyd, Sweden; 3 Faculty of Nursing, University of Alberta, Edmonton, Alberta, Canada; 4 School of Nursing and Midwifery, Deakin University, Melbourne, Victoria, Australia; 5 Cabrini-Deakin Centre for Nursing Research, Cabrini Health, Melbourne, Victoria, Australia; 6 Faculty of Medicine, University of Calgary, Calgary, Alberta, Canada

## Abstract

**Background:**

This project is part of the Translating Research in Elder Care (TREC) program of research, a multi-level and longitudinal research program being conducted in 36 nursing homes in three Canadian Prairie Provinces. The overall goal of TREC is to improve the quality of care for older persons living in nursing homes and the quality of work life for care providers. The purpose of this paper is to report on development and evaluation of facility annual reports (FARs) from facility administrators’ perspectives on the usefulness, meaningfulness, and understandability of selected data from the TREC survey.

**Methods:**

A cross sectional survey design was used in this study. The feedback reports were developed in collaboration with participating facility administrators. FARs presented results in four contextual areas: workplace culture, feedback processes, job satisfaction, and staff burnout. Six weeks after FARs were mailed to each administrator, we conducted structured telephone interviews with administrators to elicit their evaluation of the FARs. Administrators were also asked if they had taken any actions as a result of the FAR. Descriptive and inferential statistics, as well as content analysis for open-ended questions, were used to summarize findings.

**Results:**

Thirty-one facility administrators (representing thirty-two facilities) participated in the interviews. Six administrators had taken action and 18 were planning on taking action as a result of FARs. The majority found the four contextual areas addressed in FAR to be useful, meaningful, and understandable. They liked the comparisons made between data from years one and two and between their facility and other TREC study sites in their province. Twenty-two indicated that they would like to receive information on additional areas such as aggressive behaviours of residents and information sharing. Twenty-four administrators indicated that FARs contained enough information, while eight found FARs ‘too short’. Administrators who reported that the FAR contained enough information were more likely to take action within their facilities than administrators who reported that they needed more information.

**Conclusions:**

Although the FAR was brief, the presentation of the four contextual areas was relevant to the majority of administrators and prompted them to plan or to take action within their facility.

## Background

Increasingly in Canada, a focus on how research provides value beyond the usual research outputs (*e.g.*, adding to the body of knowledge, publications) is an important part of research programs [1]. Translating Research in Elder Care (TREC) is a program of research that examines the role of organizational context in facilitating the use of best practices in residential long-term care (LTC) in the Canadian Prairie Provinces [2]. The overall goal for TREC is to improve the quality of care for older persons living in nursing homes and the quality of work life for care providers. TREC accomplishes this by building an organizational monitoring system to examine associations between organizational characteristics and use of best practices (2007 to 2012). In subsequent years, we will evaluate quality improvement interventions to facilitate use of best practices and improved quality of care (2013 to 2019). Data on the nursing home’s organizational context and staff characteristics were collected in 2008 to 2010 from healthcare aides using the TREC survey. This survey, described elsewhere [2], consists of several validated instruments and measures concepts believed to constitute organizational context as defined by the Promoting Action on Research Implementation in Health Services (PARiHS) Framework [3], including leadership, workplace culture, and evaluation (feedback processes). Additionally, it includes a number of staff health related outcomes (burnout, mental and physical health), job satisfaction, attitude toward research, aggression from residents, and assessment of best practice use [2]. Data on health and clinical outcomes for the residents in the 36 participating nursing homes were collected using the Resident Assessment Instrument-Minimum Data Set version 2.0 (RAI-MDS 2.0) [4,5]. Staff surveys were completed in the 36 nursing homes in a staggered manner, with approximately one-quarter of the sites enrolled in each calendar quarter. The first set had data collection in the quarter beginning 1 June 2008. One year later, the process was repeated with the first set having a second wave of data collected in the quarter starting 1 June 2009.

Providing feedback (results) to participating facilities and staff is a component of TREC that has evolved during the course of the program. In a pilot study conducted prior to the TREC research program, care managers in four participating nursing homes had been provided with a final report on survey findings from their units [6]. This fairly lengthy (30-page) report contained detailed results and was given to care managers in a meeting where research staff explained the content of the report. Care managers’ feedback predictably indicated that the report was too extensive and, without more in-depth interpretation, difficult to understand. In TREC, we further developed the feedback activities and included healthcare aide staff [7] and facility administrators (reported here), in a timely and meaningful way that would add value to participants’ practice or environments. The feedback project was informed by our previous experiences and Rogers’ theory Diffusion of Innovations [8]. According to Rogers, successful diffusion of an innovation (such as a feedback report) depends on four elements—the innovation, the communication channels, the time, and the social system. The time element is part of the innovation-decision process, which is described in five steps: knowledge, persuasion, decision, implementation, and confirmation, by which a person proceeds from initial knowledge of an innovation to its adoption or rejection. The research team (researchers and sector partners) worked together to utilize an integrated knowledge translation (KT) approach where users of research were involved in the research process through collaboration with researchers [9]. This approach has some similarities with participatory action research methods which has been used in previous research studies in LTC [10-12] and supports a culture of using feedback to improve performance [13]. In a recent systematic review, Jamtvedt *et al.* found that audit and feedback was an effective KT intervention in improving professional practice [14]. Archer defined effective feedback as ‘feedback in which information about previous performance is used to promote positive and desirable development’ [13]. Previous methodological research on longitudinal research design has shown that use of ’Keeping in Touch Exercises‘ (such as feedback reports in various formats) between data collection periods could motivate and engage respondents in longitudinal studies to participate in forthcoming data collection and ’Keeping in Touch Exercises‘ may support researchers to maintain response rates [15]. As the TREC program progressed, KT efforts were increasingly targeted to engage staff and facility administrators in knowledge exchange activities. These interactions with research end-users and decision-makers during the TREC program are in keeping with an integrated KT approach [16].

The aim of the present project was to develop and evaluate a KT intervention, namely feedback reports to facility administrators, and examine whether facility administrators used the information to prompt action in their facilities to support their overall efforts to increase quality of care for residents and quality of work life for staff. In this paper we report on the development and evaluation of facility annual reports (FARs) from facility administrators’ perspectives to: determine whether facility administrators’ decision to take action based on FARs varied based on their perceptions of usefulness, meaningfulness, and understandability of the FARs, and report length; and assess whether facility administrators’ decisions to take action based on FAR varied by facility demographics, specifically size of facility, owner-operator model, province, and geographic location (urban or rural). The following research questions guided the study: To what extent do facility administrators: use information from the FAR to take actions in their facility?; perceive the information to be useful, meaningful and understandable?; and perceive the report to contain adequate information?

## Methods

Facility administrators (*e.g.*, site administrators, Directors of Care) from the 36 TREC study sites in the three Canadian Prairie Provinces (Alberta, Manitoba, Saskatchewan) were invited to participate in this study. A cross-sectional survey design was used in the study.

### Development of facility annual reports (FARs)

We developed the FARs using a process that included stakeholders, investigators, and policy-makers in the TREC team and administrators in TREC study sites. We notified participating facility administrators during the first year of data collection in their settings that a FAR containing TREC survey results would be provided to each of them. This feedback report was designed to share relevant information about their facility from the TREC survey. This survey consists of approximately 200 items [17], thus, it was not possible to provide information on all scales and items; content had to be prioritized. The content and format of the FARs were determined based on feedback received earlier from facility administrators. In February 2009, we sent a brief questionnaire to administrators of the 26 TREC facilities who had thus far participated in data collection and asked them to rank order the ‘top-five’ areas from the TREC survey that would interest them. At that time, we did not have access to the RAI-MDS 2.0 data and therefore could not include resident-level data in the feedback reports. Administrators were also asked to rank order preferred formats for presentation of data, such as text, tables, bar graphs, and pie charts. Twelve facility administrators responded to the questionnaire and their top ranked areas were (in order): workplace culture, feedback processes, job satisfaction, staff burnout, leadership, and use of best practices. Preferred presentation formats were text and bar graphs. TREC investigators and policy-makers discussed these data during our regular research meetings. The final template for FARs was developed based on both facility administrator responses and recommendations from TREC investigators and policy makers.

We used the same four-page booklet format for all facilities. Policy makers and a convenience sample of facility administrators counselled us during the development of the FAR by examining and commenting on various drafts of the booklet. They recommended us to limit the amount of text and the number of tables. This advice was consistent with our previous experience in the pilot study mentioned above [6]. Due to resource constraints, we decided to produce a report with standardized format and content for each facility, based on TREC survey data from unregulated care providers (healthcare aides) about four contextual areas; workplace culture, feedback processes, staff burnout, and job satisfaction [17]. The first three elements were derived scales from the TREC survey and the last was a single item. In the TREC survey, workplace culture is defined as ‘the way we do things’ in our organization and work units, and six areas of culture are assessed: recognition, support, work life balance, development opportunity, focus on service/mission, and autonomy. Feedback processes refers to group/team performance, and are assessed according to elements of the quality improvement process, namely data access, informal data review, formal data review, action planning, performance monitoring, and benchmarking. Staff burnout was measured by the Maslach Burnout Inventory (MBI) [18]. The FAR presented findings on the MBI Emotional Exhaustion dimension, which included items such as ‘I feel burned out from my work.’ Job satisfaction (one item) explores an individual’s perception of whether they are content in their current position.

**Figure 1:**
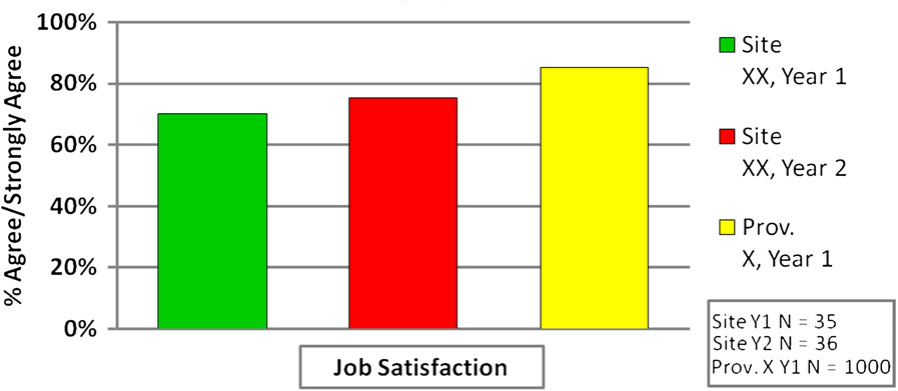
**Example of Healthcare Aide Job Satisfaction Scores presented in the Facility Annual Report. **The Job Satisfaction item explores an individual’s perception of whether they are content in their current position.

Each FAR included results of two annual data collection periods (12 months apart) for the facility along with comparative data from other TREC study sites in the same province for the first year of data collection. The first page of the report provided information about timing of the two data collection periods and sample size at each time point. The second and third pages presented the results for the contextual areas in a bar graph accompanied by brief explanatory text. An example for Job Satisfaction is presented in Figure 1. The fourth page included contact information for TREC investigators and the provincial research manager.

### Administration of the facility annual reports

FARs were prepared for quarterly distribution. Specifically, each facility administrator received the FAR within two months of completion of time two data collection. A cover letter introducing each FAR and an invitation to complete a short interview to evaluate the feedback reports were sent to facility administrators via express post.

### Data collection

To evaluate their perceptions of the FAR, we conducted telephone interviews with facility administrators three to six weeks after sending the FARs. Interviews were conducted by two researchers using a structured interview guide, consisting of nine questions with fixed response alternatives. Four of these nine questions had an open-ended follow-up question. Administrators were asked the extent to which they found the presentation of each of the four contextual areas (workplace culture, feedback processes, job satisfaction, and staff burnout) meaningful, understandable, and useful. They were asked to rate their responses using a four-point Likert scale, from 1 = very low extent to 4 = very high extent. Administrators were also asked whether the FAR prompted them to implement any changes within their facility. They were encouraged to elaborate on their responses, for example, whether they wanted to have information about other areas, not currently included in the FAR, in the future. Responses were recorded on the interview guide. Interviews took approximately 20 to 30 minutes to complete. In addition to the interview data, facility administrative data on areas such as number of beds (size of facility) and owner-operator model were used in the analyses. These latter data are routinely collected in the TREC program.

### Ethical considerations

Ethical approvals for this study were obtained from Health Research Ethics Boards from the University of Alberta, University of Calgary, University of Saskatchewan, and the University of Manitoba and the operational review boards (where applicable) for all facilities participating in the study.

### Data analysis

Data were double entered into a PASW statistics database. Data were analyzed using PASW^©^ version 18.0 [19]. Descriptive statistics were used to summarize the data. ANOVA tests and Chi-Square tests (Fisher’s Exact when appropriate) were used to assess the differences in mean values and between proportions. We used content analysis to code responses to the open ended questions.

## Results

Of the 36 facility administrators, 31 participated in the study. Because one administrator was responsible for two facilities, data were obtained from 32 nursing homes (response rate 89%). Of the 32 nursing homes, 15 were in Alberta, 11 were in Saskatchewan, and 6 were in Manitoba. See Table 1 for a summary of facility demographics on ownership model, facility size and geographic location.

**Table 1:** Facility demographics

**Demographic**	**N = 32 nursing homes**
**Province**	
Alberta	15
Saskatchewan	11
Manitoba	6
**Ownership model**	
Public	11
Private for profit	7
Voluntary	14
**Facility size**	
Small (35 to 79 beds)	13
Medium (80 to 120 beds)	7
Large (>120 beds)	12
**Geographic location**	
Urban	27
Rural	5

Six administrators reported that they had taken actions in their nursing homes in response to the FAR. Actions included development of a staff survey about triggers of stress in the workplace (with the goal of developing a support program), use of information in the facility’s business plan, implementation of a log book to improve communication processes with staff, installation of additional ceiling track lifts to improve staff resources (in an effort to reduce staff burnout), and comparison of FAR data with results of an internal facility survey conducted by the quality committee. One administrator indicated that the FAR revealed a lack of communication among staff. Another administrator reported that staff lacked social support after experiencing stressful situations at work.

A further 18 administrators who reported that they intended to take actions as a result of the FAR were planning the following activities: conduct an internal employee satisfaction survey and job evaluation to compare findings with the FAR, invite a speaker to talk with staff about grief (in an effort to reduce workplace staff burnout), and conduct performance appraisals (feedback processes). Three administrators were planning to incorporate FAR findings into their facility’s business plan, while four administrators were gathering more information to inform decisions about actions to improve workplace culture.

The majority of administrators considered the FAR useful. Mean values for usefulness of the four contextual areas ranged between 3.34 and 3.06 (range 1 to 4) (Table 2). Three administrators commented that findings reported in the FAR confirmed observations about areas such as staff burnout. Three administrators indicated the FAR was useful because it confirmed findings from previous staff surveys at their facility (*e.g.*, staff satisfaction). Most administrators who indicated the FAR was useful found comparisons between years one and two of data collection and within-province comparisons of study facilities useful for benchmarking. Five administrators found the staff burnout information less useful due to lack of knowledge on this area. Twenty-six administrators reported sharing the FAR with the Chief Executive Officer, care managers, the management team, and the staff. Seven administrators had shared the information with persons outside the facility, such as those at the corporate/regional office.

**Table 2:** Administrators’ scoring of the facility annual report

		**n**	**Workplace culture m (SD)**	**P-value**	**Feedback processes m (SD)**	**P-value**	**Job satisfaction m (SD)**	**P-value**	**Staff burnout m (SD)**	**P-value**
**Usefulness**										
	Total sample	32	3.34 (0.70)		3.06 (.76)		3.31 (0.74)		3.16 (0.77)	
Taken actions	Yes	6	3.50 (0.55)	0.577	3.50 (0.55)	0.281	3.50 (0.55)	0.377	3.17 (0.75)	0.803
	Not yet	18	3.39 (0.50)		3.00 (0.69)		3.39 (0.61)		3.22 (0.55)	
	No	8	3.13 (1.13)		2.88 (0.99)		3.00 (1.07)		3.00 (1.19)	
**Meaningfulness**										
	Total sample	32	3.44 (0.62)		3.13 (0.71)		3.44 (0.62)		3.23 (0.72)	
Taken actions	Yes	6	3.50 (0.55)	0.628	3.33 (0.52)	0.692	3.50 (0.55)	0.628	3.17 (0.75)	0.766
	Not yet	18	3.50 (0.51)		3.11 (0.76)		3.50 (0.62)		3.33 (0.77)	
	No	8	3.25 (0.89)		3.00 (0.76)		3.25 (0.71)		3.13 (0.64)	
**Ease of Understanding**										
	Total sample	32	3.44 (0.62)		3.25 (0.80)		3.56 (0.50)		3.41 (0.62)	
Taken actions	Yes	6	3.50 (0.89)	0.628	3.50 (0.55)	0.686	3.50 (0.55)	0.493	3.50 (0.55)	0.761
	Not yet	18	3.50 (0.51)		3.22 (0.81)		3.50 (0.51)		3.33 (0.59)	
	No	8	3.25 (0.89)		3.13 (0.99)		3.75 (0.46)		3.50 (0.76)	

1 = to very low extent to 4 = to very high extent.

ANOVA test.

Facility administrators scoring of the FARs usefulness, meaningfulness and ease of understanding for each of the four areas (workplace culture, feedback processes, job satisfaction and staff burnout) and a comparison of facility administrators scoring (mean value) of these areas based on taken actions in facility due to FAR.

The majority of administrators found the four contextual areas in the FAR meaningful. Mean values for meaningfulness ranged from 3.44 to 3.13 (Table 2). Twenty-two administrators indicated they would like more information from the TREC survey on areas such as aggressive behaviours of residents, information sharing, and overall job satisfaction at the facility. Concerning areas of interest not included in the TREC survey, administrators suggested quality of care indicators, staffing levels, and time utilization would be important in future FARs.

Information contained in the FAR was perceived to be understandable by nearly all administrators. Mean values for ease of understanding ranged from 3.56 to 3.25 (Table 2). Of the 31 administrators, eight indicated the FAR was too short and they wanted more information. Three administrators indicated that the workplace culture items were unclear, in particular, support. The type of support the staff needed was not clear to these administrators. To make the FAR more clear and understandable, a few administrators suggested including a definition for each of the four contextual areas and/or the survey questions relating to each area.

Administrators who perceived that the FAR contained enough information were more likely to take actions within their facilities than the administrators who reported that they needed more information (Table 3). We found no significant differences in administrator reported mean values for the usefulness, meaningfulness, and understandability of the FARs based on whether they had decided to take action, were planning to take action or had decided not to take action (Table 2).

**Table 3:** Administrators’ perceptions whether the FAR contained enough information and their decision to take actions

		**n**	**Yes, have taken actions n (%)**	**Not yet n (%)**	**No actions n (%)**	**P-value**
Length of FAR	Enough information	24	6 (25)	13 (54)	5 (21)	0.0177
	More information	8	0	5 (62.5)	3 (37.5)	

Fisher exact test.

We explored whether facility administrators’ decisions to take actions based on the FAR varied by facility demographics (size of facility, owner-operator model, province, urban versus rural). Five of the six administrators who decided to take action worked in small facilities (Table 4). The remaining administrator worked in a large facility. In regards to owner-operator model, two administrators from each of the three groups (public not for profit, private for profit, and voluntary not for profit) had taken actions. We found that in one of the three provinces no administrators had taken actions based on the FAR; in the other two provinces, three administrators in each province reported that they had taken action. Five of these administrators worked in urban facilities. We did not find any statistically significant differences between the proportions of administrators who reported taking action based on facility demographic variables (Table 4).

**Table 4:** Comparison of facility demographics and administrators’ reported decisions to take actions

		**n**	**Yes, have taken actions n (%)**	**Not yet taking actions n (%)**	**No actions n (%)**	**P-value**
Size of facility	Small	13	5 (38)	7 (54)	1 (8)	0.1144
	Medium	7	0	5 (71)	2 (29)	
	Large	12	1 (8)	6 (50)	5 (42)	
Owner operator model	Public	11	2 (18)	7 (67)	2 (18)	0.5596
	Private for profit	7	2 (29)	2 (29)	3 (43)	
	Voluntary	14	2 (14)	9 (64)	3 (21)	
Province	1	15	3 (20)	8 (53)	4 (27)	0.7914
	2	11	3 (27)	6 (55)	2 (18)	
	3	6	0	4 (67)	2 (33)	
Geographic location	Urban	27	5 (19)	15 (56)	7 (26)	1.000
	Rural	5	1 (20)	3 (60)	1 (20)	

Fisher exact test.

## Discussion

In this discussion we present lessons learned during the development and distribution of the FAR, and suggestions for future research on providing feedback to administrators.

### Which areas should be presented in a feedback report?

Previous research has stressed the importance of involving decision makers in the research process to increase the likelihood of uptake of research findings in practice [20]. There is little literature with detailed information on how to actually provide feedback efficiently [21]. Building on the integrated KT model used in TREC, we engaged all stakeholders to find out which items and scales from the TREC survey should be presented in the FAR. The findings of the survey of administrators provided their views on which contextual areas they rank ordered as most important and their preferred presentation formats. Administrators rank ordered two contextual areas (leadership and use of best practice) highly for inclusion in the FARs. However, the research team considered it premature to present these two contextual areas without an opportunity to do face-to-face debriefing and so excluded these areas. Staff perceptions of leadership are often a sensitive topic and not well suited to a short feedback report or one without face-to-face interaction, particularly for those who may have poorer results. Without more detailed discussion about the meaning of results, administrators might have difficulty determining what if any actions might be warranted and if so, which might be most relevant.

In developing the FAR, resource constraints required that we present the same four contextual areas to all administrators. From the interviews, the majority of administrators desired information on additional areas from the TREC survey, such as resident aggressive behaviours towards staff, information sharing between staff, and overall job satisfaction. Some administrators also wanted to have quality of care indicators for future FARs. One basic principle for successful feedback is to tailor the feedback to the recipient’s needs and understanding; in this case to enhance quality of care for the residents and quality of work life for staff [13]. Thus, another approach to selecting contextual areas for the FAR is to provide each administrator with individualised facility feedback, tailored to her/his information preferences. Individualized and customized feedback has been shown to be more useful to clinicians for improving quality of care [22]. This approach is likely also more useful in informing administrator decision making, enabling targeting of contextual areas in which potential deficiencies in quality of care for residents and quality of work life for staff have been identified. The drawback of this approach is that the time required to create individualised feedback reports is increased, and thereby report production more expensive. However, if administrators find the feedback useful and take action based on information contained in the feedback report, the increased cost can be justified and built in *a priori*. A useful future study might examine whether a tailored feedback report from the perspective of administrators and managers prompts a greater percentage of administrators to take action in their facilities compared with a generic template feedback report.

### What does enough information actually mean?

Administrators who reported that the FAR contained enough information were more likely to have taken actions when compared with administrators who needed more information. We did not ask administrators to elaborate on the reasons why they reported that the FAR contained enough information or not. Some of them indicated that including the survey question might have helped them to understand the responses better.

When the FAR was mailed to administrators, we did not provide an opportunity for more detailed information about its content, such as an information session or detailed individual outreach. At the time of the follow-up phone call, the interview became the opportunity for the administrators to ask their questions about the content. Several administrators requested more information, for example, about the meaning of feedback processes and staff burnout. These two areas were also scored lowest by the administrators in relation to usefulness, meaningfulness, and ease of understanding (Table 2). While the systematic review by Jamtvedt *et al.* did not provide evidence that audit and feedback combined with other interventions, such as educational meetings or outreach, were more effective than audit and feedback alone [14], our interviews revealed that it was helpful and valuable for the administrators to speak with researchers in order to obtain more in-depth knowledge about included areas. Educational support during feedback to teams has been identified as a key factor to facilitate learning and change [23].

The Decision Innovation Process from Roger’s theory is helpful in interpreting findings from this study [8]. For the administrators who reported taking action based on the FAR, the report could have been the ‘tipping point’ that led them to take action. Some of these administrators reported that the FAR confirmed findings of staff surveys conducted in the facility prior to the TREC survey, which prompted them to take action. For them, the FAR contained information that was timely. For administrators who reported that they were considering taking action (these administrators were likely to ‘be in’ the persuasion phase), most were collecting additional information to help inform their decisions. This finding aligns with planned change theories that state that decisions on changing behaviour or other actions will occur when feedback fulfils the needs of the recipient to achieve a desired goal [13].

Our findings suggest that future research on feedback should provide opportunities for face-to-face conversations with administrators in areas such as: describing more about the content of presented areas and data; discussing the importance of this area in relation to quality of care for residents and quality of work life for staff; and facilitating decisions about what actions could be taken by facility administrators. Future research should also explore, from the perspective of administrators and managers, what constitutes the optimal amount of information in a feedback report to inform decision making.

### Sharing the FAR in the facility

Twenty-six administrators reported that they had shared the information with their management group or team in the facility. Some of them had also shared the FAR with front-line staff. In the information letter which was sent together with the FAR we did not recommend that the administrators share the FAR or take any action. The FAR was looked upon by the investigators as the administrator’s privileged report, and it was up to the administrator to decide what to do, with whom, and when. Although we received responses that some of the information in FAR was a bit unclear, the administrators did share the FAR with care managers and staff. We believe that the sharing of the FAR is an important step in the process of enhancing quality of care for residents and quality of work life for staff. Several researchers have suggested that leadership is crucial to successful quality improvement and the implementation of research findings in practice [24-28]. Important aspects of leadership are to facilitate communication and teamwork, and to create an open and blame-free culture [28]. It is also important to involve policy makers and decision makers in the research process [20], administrators need to involve frontline managers and staff in quality improvement efforts in nursing homes [26,28]. It appeared that the majority of administrators had started a process to involve managers and staff at the time of our interviews, and future research will investigate initiatives to enhance the quality of care and quality of work life for staff. Further studies could also examine administrators’ perceived needs for and access to support the uptake of research findings and quality improvement in their facility.

### Limitations

We note some study limitations. First, although nearly all administrators of the 36 facilities in the TREC research program participated in this study (response rate was 89%), the sample size was small (n = 31). This limited our statistical analysis because we were unable to conduct advanced statistical modeling. We conducted ANOVA analysis for some of the research questions however the small sample means we have greater risk for type I error. The findings from these analyses must be interpreted with caution. Second, interviews were conducted by phone using a structured interview guide with several questions including fixed response alternatives. This was done in order to keep the interviews short, given administrators’ limited time. However, this approach limited the opportunity for in-depth exploration of some areas. For example, respondents’ perceptions of what was ‘enough’ information in the report. Third, potential for social desirability bias may have led to overestimation of the FAR’s usefulness. In future research, investigators should include interviews with other staff, such as care managers to add a variety of perspectives and enrich the evaluation of the feedback reports. Furthermore, a larger sample of facility administrators would be needed to conduct advanced statistical analysis.

## Conclusions

Although the FAR was a short four-page brochure with brief text and tables the presentation from the four contextual areas made sense for the majority of administrators and prompted them to plan or to take actions within their facility. The findings of the FAR project have important implications for providing feedback to facility administrators. First, clarity is needed on data to include in feedback reports, and how to tailor feedback to the needs of the administrators. In particular, relevance of content and level of detail may potentially influence the likelihood of findings being used to inform change. Second, means of distribution should be considered. Just sending a feedback report without opportunity for support or discussion with a knowledgeable person will likely reduce its usefulness. Finally, expectations on the extent and type of actions that administrators will undertake due to the feedback report must be realistic. The report will be one information source among many that inform decision-making processes.

## Abbreviations

TREC: Translating Research in Elder Care; FAR: facility annuals report; LTC: long-term care; KT: knowledge translation; MBI: Maslach Burnout Inventory.

## Competing interests

The authors declare that they have no competing interests.

## Authors' contribution

AMB designed the study, provided leadership for the study and lead the manuscript development. LAC assisted in data collection and analysis, and manuscript development. AMH contributed to the design of the feedback reports and manuscript development. GGC is provincial site lead for the TREC research program in Alberta. PN is co-lead investigator for project one of the TREC research program. CAE is the principal investigator for the TREC research program and conceived the FAR study. CAE, PN and GGC participated in securing the funding for the TREC research program. CAE, GGC and PN provided commentary on the final submitted manuscript. All authors read and approved the final submitted manuscript.
